# Aging microglia

**DOI:** 10.1007/s00018-023-04775-y

**Published:** 2023-04-21

**Authors:** Ignazio Antignano, Yingxiao Liu, Nina Offermann, Melania Capasso

**Affiliations:** Deutsches Zentrum für Neurodegenerative Erkrankungen (DZNE), Bonn, Germany

**Keywords:** Microglia, Aging, Neuroinflammation, RNAseq, Human microglia, Senescence

## Abstract

Microglia are the tissue-resident macrophage population of the brain, specialized in supporting the CNS environment and protecting it from endogenous and exogenous insults. Nonetheless, their function declines with age, in ways that remain to be fully elucidated. Given the critical role played by microglia in neurodegenerative diseases, a better understanding of the aging microglia phenotype is an essential prerequisite in designing better preventive and therapeutic strategies. In this review, we discuss the most recent literature on microglia in aging, comparing findings in rodent models and human subjects.

## Introduction

Microglia are the resident immune cells of the central nervous system (CNS), a tissue-resident macrophage population with specific characteristics to support the CNS environment and health.

Microglia have a mesodermal origin and originate from yolk-sac progenitors during embryogenesis; after their early migration and proliferation, they colonize the CNS and self-renew throughout the lifespan [1, 2], with no or perhaps little contribution from bone marrow-derived monocytes [3], as previously hypothesized [4].

Microglia perform a variety of critical functions; (a) they support neurogenesis and ensure correct neuronal circuitry by pruning synapses [5]; (b) phagocytose apoptotic neurons [6]; (c) defend against infectious [7] and non-infectious insults [8]; (d) produce extracellular matrix (ECM) components and control its remodeling by secreting ECM-degrading enzymes [9]; (e) maintain myelin health [10]; (f) and remove extracellular protein aggregates [11], which accumulate in neurodegenerative diseases [12].

Homeostatic adult microglia have a highly ramified morphology, with extended and arborized processes and a small soma. However, when responding to stimulation or during aging and CNS pathology, their morphology changes [13], as first observed by Pío del Río-Hortega more than 100 years ago [14].

With age, microglia alter their function, morphology and phenotype; however, there are still many gaps in our knowledge of how microglia age, especially if the aging process affects human and rodent microglia in a similar fashion. Age is the greatest risk factor in many neurodegenerative diseases, in which microglia play a crucial role [15, 16]; therefore, it is important to better understand microglia alterations in aging, before we can develop new preventive measures and new curative therapies.

The present article will review our current knowledge on aging microglia, with a special focus on more recent literature, highlighting points that remain to be further elucidated. We will start by reviewing the literature on microglia from animal models and follow with a review of human studies, identifying similarities and discrepancies.

## Phenotype of aged rodent microglia

The majority of studies on aged microglia have been carried out in rodent models. In the early nineties, Hugh Perry and coworkers showed that aged rat microglia had an altered morphology with shortened and less complex branching, especially in the white matter, the part of the CNS containing nerve fibers surrounded by myelin sheets [17]. Furthermore, they reported that, in aged brains, a proportion of microglia expressed higher levels of activation markers, such as MHC II, CD45 and CD4; however, these markers were equally distributed between white and gray matter [17]. These observations, together with previous evidence that aged microglia had increased inclusions, indicative of greater phagocytosis activity [18, 19], led to the suggestion that microglia or at least a subset had an activated phenotype in aging. Further confirmation of these findings over the years corroborated the idea that aged microglia have a primed phenotype [20], not a more inflammatory state at steady state but a propensity to respond more strongly to stimulation, due to their downregulation of homeostatic and inhibitory genes (CD200, CX3CL1, CD47, and Sialic acid) and upregulation of stimulatory ones (MHC, CD86, CD68, CR3, pattern recognition receptors such as TLRs and Clec7a) [21, 22]. These alterations have been observed across different regions in the aged brain, with more pronounced changes in white matter-resident microglia, indicating that they are responding to myelin-related alterations in aging [23].

Although aged microglia respond to an inflammatory challenge (like peripheral LPS administration) by producing higher levels of pro-inflammatory cytokines such as TNF-α, IL-1β, and IL-6 compared to young counterparts, anti-inflammatory signatures have also been observed [24–27]. Nonetheless, it has been shown that, although aged microglia still expressed IL-10 and IL4R-α at levels comparable to juvenile counterparts, these cytokines failed to reduce inflammation due to lower cellular responsiveness [28]. As a consequence, aged microglia did not return to homeostasis and the prolonged inflammatory state exacerbated cognitive impairment, sickness and depressive-like behaviors to levels normally observed in neuroinflammation settings [24, 26, 28].

The increased ability of aged microglia to respond to stimulation was confirmed by RNA sequencing studies [29, 30], which also found an upregulation of neuroprotective pathways [29] and cytoskeletal alterations [30], in line with morphological studies showing altered morphology of aged microglia. In addition, a co-expression analysis of primed microglia from healthy aged animals and models of accelerated aging and neurodegeneration identified a common expression profile characterized by an upregulation of immune receptors (*Clec7a*, *Itgax*, and *Cxcr4*), phagosome- and lysosome-related genes (*Axl* and *Lgals3*) and lipoprotein *Apoe* [31]. This signature was distinct from the acute inflammatory gene set induced in LPS-treated animals, in which genes related to the NF-κb pathway and Toll-like and NOD-like receptor signaling were highly enriched. Furthermore, the comparison between aged and diseased mice revealed a specific expression profile in the aging group, characterized by an enrichment of genes related to ribosomal proteins and interferon (IFN) α/β signaling [31].

In addition to their heightened responsiveness to stimulation and altered morphology, further alterations have been described in aged microglia, such as their reduced ability to scavenge the brain parenchyma to remove neuronal debris, apoptotic bodies and secreted proteins through phagocytosis [32, 33]. One of the responsible factors is CD22, a negative regulator of microglia phagocytosis [34, 35], whose expression in microglia in the CNS increases with age. Genetic ablation or inhibition through anti-CD22 injection in mice robustly promoted the clearance of myelin debris, rescued the age-related microglial signature and improved cognitive functions [34]. Furthermore, these changes were accompanied by increased microglia surveillance and motility and a restoration of microglia homeostatic morphology, as observed via two-photon imaging [35]. Notably, CD22 is also expressed and secreted by mouse neurons, exerting an inhibitory role on microglia in soluble form [36]. In a similar fashion, soluble CD22 is released by oligodendrocytes in the human brain, whereas human microglia do not express it. Nonetheless, binding of soluble CD22 to insulin-like growth factor 2 receptor (IGF2R) expressed by microglia altered their lysosomal protein trafficking in Niemann Pick type C [37]. It remains to be elucidated if a similar increase in soluble CD22 happens in human brains in aging and its effect on microglia phagocytosis.

As mentioned earlier, an important microglia function is to phagocytose synapses, a process essential during development in order to ensure correct neuronal connectivity [38]. Nonetheless, synaptic pruning happens at low level throughout the lifespan, as a mechanism to ensure neuronal plasticity: indeed, microglia-mediated synaptic pruning is neuronal-activity dependent [5, 39]. It remains to be completely elucidated if microglia-mediated synaptic phagocytosis is responsible for the decline in synaptic loss that correlates with cognitive decline, as observed in aging human brains [40] and possibly also mouse brains [41], although there are discordant reports [42]. Nonetheless, studies from mouse models have helped shed light on the mechanisms mediating synaptic phagocytosis, which is regulated by synapse tagging with “eat me” signals that are recognized by microglia. The main signals are constituted by complement proteins C3 and C1q, which tag synapses through phosphatidyl serine externalization or potentially additional molecules [43]. Complement-decorated synapses are then recognized by microglia through complement receptors [5, 44]. Indeed, C3 deficiency was shown to protect mice from age-related hippocampal decline [41] and C1q deposition and production in aging mouse brain is greatly upregulated [45]. Additional studies have indicated that C1q and C3 are implicated in synaptic loss in mouse models of Alzheimer’s disease and inflammation [44, 46–48]**.** Nonetheless, it remains to be fully elucidated if aging microglia contribute to excessive synaptic phagocytosis through increased production of complement components (which appears more pronounced for C1q than C3 [45, 46]); decreased recognition of “don’t eat me” signals, as reported in neurodegeneration [49]; increased expression of complement receptors, such as CD11b [27]; increased overall phagocytosis by certain microglia subsets [50] or a combination of all different factors.

### Metabolism and oxidative stress in aged microglia

A well-tuned energy metabolism is essential for immune cells to maintain homeostasis and meet the demand of bio precursors needed to mount an efficient immune response [51]. Aging, however, is characterized by the deterioration of nutrient-sensing networks and a collapse of mitochondrial efficiency and integrity [52]. Indeed, aged microglia also present an altered metabolism and downregulate oxidative phosphorylation genes [29]. Possibly in response to altered energy metabolism or because of an altered balance of growth factors in the brain environment, aged microglia also possess heightened levels of mammalian target of rapamycin (mTOR) signaling [27]. mTOR is a critical node that regulates cell growth and metabolism in all cell types and its inhibition is linked to increased longevity [53]. In aging microglia, increased mTOR signaling provides a further layer of regulation of the primed phenotype, by enhancing mRNA translation [27]. In this study, we revealed that aged microglia upregulated mTOR complex 1 (mTORC1) signaling and downstream mRNA translation, through the 4EPB1-EIF4E axis [27]. Increased mTOR-dependent phosphorylation of 4EBP1 resulted in higher translation, and, therefore, higher protein levels, of inflammatory receptors and cytokines in aged microglia compared to younger counterparts, an effect that could be reduced by loss of Rheb1, the upstream positive regulator of mTORC1. Interestingly, as reported by Holtman et al. [31], microglia cells characterized by increased mTOR signaling had higher transcript levels of ribosomal genes [27].

Metabolic changes might also directly cause low-level inflammation in aged microglia, such as the upregulation of pro-inflammatory prostaglandin E2 (PGE2) signaling [54]. Through the prostaglandin E2 receptor 2 (EP2), PGE2 promoted the sequestration of glucose into glycogen via the AKT–GSK3β–GYS1 pathway, leading to a reduction of glucose flux, lower mitochondria respiration and ATP production. This energy-depleted state resulted in decline of de novo NAD + synthesis, which impaired the NAD-dependent Sirt3-mediated deacetylation of mitochondrial complex II subunits, leading to lower succinate dehydrogenase activity [55]. Consequently, the accumulation of succinate, a TCA cycle metabolite, stabilized the activity of hypoxia-inducible factor 1α (HIF-1α), an activator of pro-inflammatory cytokines [54]. Blockade of PGE2 (genetically or pharmacologically) reverted this phenotype towards a more anti-inflammatory signature in peripheral macrophages and microglia, reduced the expression of pro-inflammatory cytokines in the blood and hippocampi of aged animals and caused an amelioration of hippocampal plasticity and memory function [54]. Notably, the peripheral inhibition of the EP2 receptor with a brain-impermeant EP2 antagonist in aged animals yielded a similar outcome, indicating that age-associated brain inflammation and cognitive decline is not an irreversible program and can be modified by intervening on peripheral cells.

**Fig. 1:**
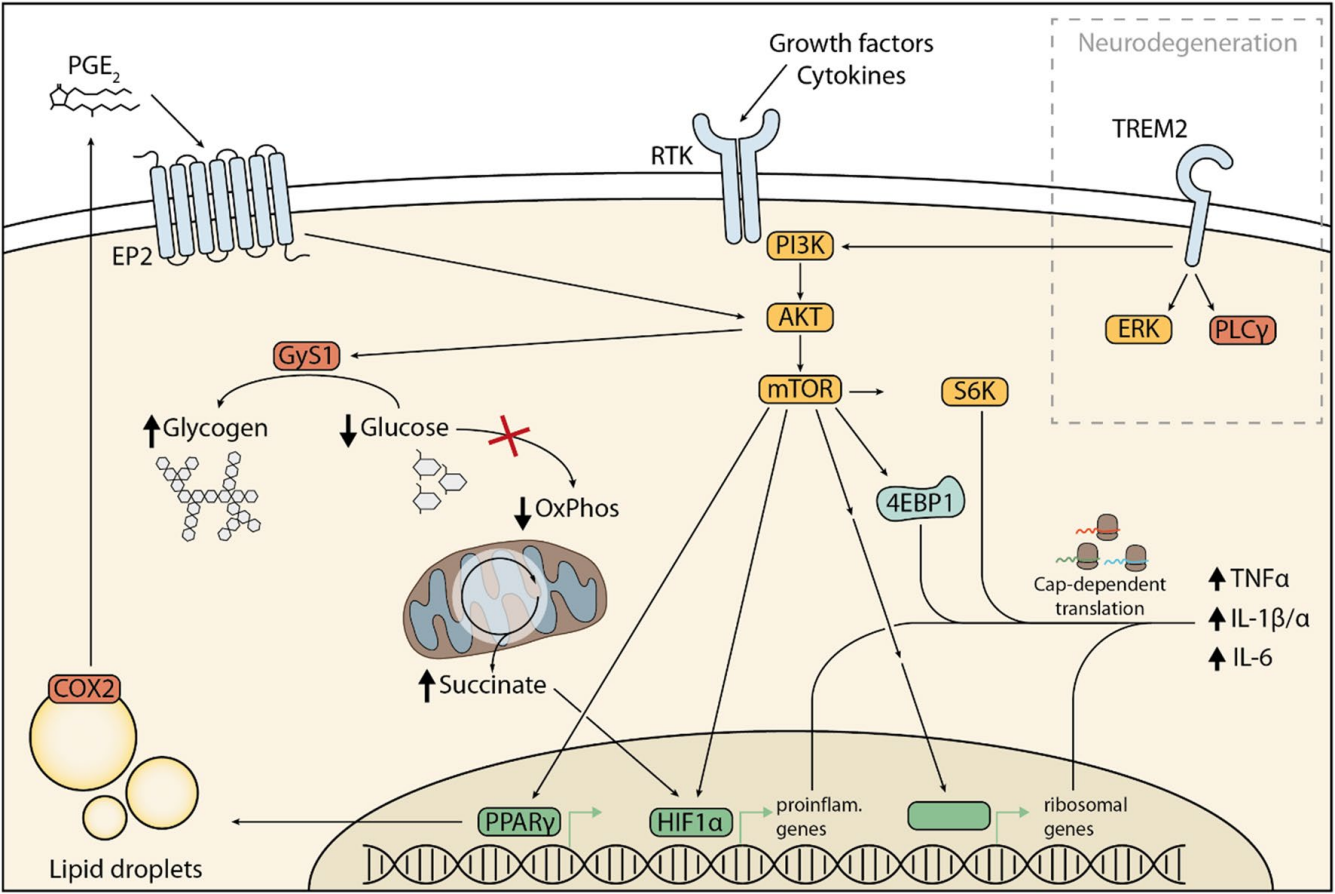
Signaling pathways altered in aging microglia. Aging is characterized by changing in metabolism, such as increased levels of eicosanoids, such as prostaglandin E2 (PGE2) [54], growth factors and cytokines that lead to the activation of the PI3K-AKT-mTOR signaling pathway in microglia [27]. AKT activation promotes sequestration of glucose into glycogen, causing energy depletion and accumulation of succinate [54]. Succinate is known to stabilize the transcription factor HIF1a, which is also induced by mTOR signaling [150] and promotes transcription of pro-inflammatory genes [54]. In parallel, mTORC1 induces transcription of ribosomal genes through multiple mechanisms [151–153]. Collectively, this results in increased translation of pro-inflammatory factors such as TNF, IL-1b, and IL-6 in aged microglia [27]. Downstream of mTOR, the transcription factor PPARγ, among others, promote lipid droplet formation in myeloid cells [154], suggesting a potential similar mechanism in microglia. COX2, which is located at the droplet membrane, in turn produces more PGE2, leading to a feed-forward loop [154]. The PI3K-AKT-mTOR pathway is activated by TREM2 and is required for microglia ability to respond to amyloid-β deposition [60]. TREM2 variants resulting in reduced expression are associated with neurodegeneration [61, 62].

More generally, lipid metabolism is altered in aged microglia. It has long been known that aged microglia accumulate inclusions in their cytoplasm, such as lipid droplets [18]. Nonetheless, new studies have described the functional consequences on the aged phenotype. Transcriptome analysis of the so-called Lipid droplets-associated microglia (LDAM) showed a severe phagocytosis deficit and an increased production of reactive oxygen species (ROS) and inflammatory cytokines, associated with a signature that partially overlapped with that of microglia from LPS-treated mice [56]. Interestingly, genes regulating lipid droplet formation in LDAM were also found upregulated in models of neurodegeneration [56], suggesting that aged microglia may partially share a common signature or acquire a phenotype that might accelerate neurodegeneration. A schematic summary of metabolic changes in aging microglia is provided in Fig. 1.

### Microglia heterogeneity in aging

The emergence of novel high-throughput technologies has shed new light on microglia heterogeneity, which appears to be significantly influenced by microenvironmental cues.

A first comprehensive study of the regional heterogeneity of aged microglia came from Barry McColl and coworkers [57]. In this study, the authors carried out a transcriptome study of microglia from different brain regions, across the adult lifespan of mice. All regions were characterized by a slight age-dependent increase of immune-regulatory genes, nonetheless, the greatest differences were observed in cerebellar and hippocampal microglia. In the cerebellum, aged microglia upregulated genes involved in the interferon pathway, such as regulatory factors (*Irf7*, *Stat2*, and *Oals2*), effectors in antiviral defense (*Sp100*, *Csprs*, *Isg20*, *Ifit*, *Bst2*, and *Zbp1*) and activating immune receptors (*Cd300ld*, *Trem1*, *Sirp1a*, *Cd200r4*, and *Cd200lb*). Conversely, with aging, hippocampal microglia downregulated genes involved in cell adhesion and motility, interaction with matrix components and other extracellular ligands (*Cd36*, *Cd93*, *Pf4*, and *Lyve1*), endocytosis/phagocytosis (*Arhgef3*, *Dab2*, *Itsn1*, and *Vav3*) and, finally, mannose receptor gene *Mrc1* and the MHC II genes *H2-Aa* and *H2-Ab1*. The authors hypothesized that these gene alterations suggest a potential “disengagement” of aged hippocampal microglia with their environment, possibly explaining why the hippocampus is vulnerable to age- and disease-related deposition of misfolded proteins [57].

The optimization of new technologies such as single-cell RNA sequencing (scRNAseq) has resulted in a number of new studies on microglia in healthy aging and disease, which have revealed a complex and dynamic phenotypic landscape. In Hammond et al. [50], the authors described the presence of five clusters that emerged when comparing microglia from 2-months old and 18-months old mice. Two subsets, old activated 1a and b (OA1a and OA1b), had a slightly altered phenotype compared to young microglia; however, the number of cells occupying these states were similar in both adult and aged animals. Conversely, the two clusters named OA2 and OA3, progressively expanded with age in the absence of pathology, along with a monocyte and macrophage cluster (Mono/Mac), both indicative of a shift towards a more inflammatory or more primed phenotype [50]. The OA2 subset showed upregulation of inflammatory genes, such as *Ccl3*, *Ccl4*, *Tnf*, *Il1b*, *Tlr2*, *Spp1*, lipid metabolism and phagocytic genes (*Apoe*, *Lpl*, *Cst7*, and *Gpr84*), as well as cellular stress response markers (*Id2* and *Atf3*). The cluster OA3, instead, was characterized by interferon-associated genes, including *Ifi27l2a*, *Ifitm3*, *Rtp4*, *Ifit3* and *Oasl2*, which may play a role in modulating inflammation [50]. In another scRNAseq study, Sala Frigerio et al. drew similar conclusions, albeit analyzing only microglia from cortex and hippocampus across the mouse lifespan [58]. The authors found the majority of microglia clustered in two subsets, homeostatic 1 microglia and homeostatic 2 microglia (H1M and H2M), characterized by high expression level of homeostatic markers *Cx3cr1*, *Tmem119* and *P2ry12*. Both clusters were equally detected at every time point investigated; however, H2M showed a decrease by ~ 10% at 21 months old [58]. In addition, two cell clusters were found to expand with age, partially overlapping with those previously described in Hammond et al. [50]. The first one was defined as activated response microglia (ARM), characterized by increased expression of genes involved in inflammatory processes (*Cst7*, *Clec7a*, and *Itgax*), along with MHC II-associated genes (*Cd74*, *H2-Ab1*, *H2-Aa*, *Ctsb*, and *Ctsd*) and genes involved in tissue regeneration (*Spp1*, *Gpnmb*, and *Dkk2*). A second cluster, named IFN response microglia (IRM), was characterized by genes of the type I interferon-response pathway, similarly to the OA3 cluster described by Hammond et al. [50]. In both studies, the aged microglia states appeared to be the result of a progressive expansion of clusters that were detected at low levels in younger animals. Sala Frigerio et al. further showed that they temporally originated from the homeostatic microglia cluster, which comprised the majority of microglia in young adults [58]. Indeed, despite the expansion of these clusters in aging, the “activated” clusters only constituted a small fraction of the whole microglia population, with the inflammatory responsive microglia (OA2 and ARM) representing the highest percentage of cells accumulating in aging.

The activated phenotype of OA2 and ARM microglia shared some similarities to the microglia phenotype defined as damage-associated microglia (DAM) by Ido Amit and coworkers in models of neurodegeneration [59]. The authors showed that DAM originate from a homeostatic subset through an intermediate state, which expanded as a result of disease progression in 5xFAD mice, an Alzheimer’s disease (AD) mouse model of amyloid-β deposition and neurocognitive alterations. DAM were characterized by a suppression of the homeostatic microglia signature (*Tmem119*, *P2ry12*, *P2ry13*, *Cx3cr1*, *Cd33 or Siglec-3*, and *Csf1r*) and increased expression of cell surface receptors (*Itgax*, *Trem2*, *Tyrobp*, *Clec7a*, and *Lilrb4*), tetraspanins (*Cd9* and *Cd63*), MHC II (*Cd74*), cytokines (*Csf1*, *Il1b*), chemokines (*Ccl3*, *Ccl4*, and *Ccl6*), growth factors (*Igf1*), as well as molecules involved in lipid metabolism, phagocytosis, lysosome functions (*Apoe*, *Lpl*, *Axl*, *Cst7*, *Ctsb*, *Ctsd*, *Ctsl*, and *Ctsz*), and tissue remodeling (*Gpnmb*, *Spp1*, adn *Timp2*). Notably, the transition from homeostatic to DAM occurred in a two-step mechanism. First, homeostatic microglia switched to a TREM2-independent intermediate state (named stage 1 DAM), defined by upregulation of AD risk factors (*Apoe*, *Tyrobp*, *B2m*). Then, when mice reached an older age, microglia progressed towards a TREM2-dependent state (defined as stage 2 DAM), characterized by an additional signature of higher expression of lipid metabolism and phagocytic genes (*Trem2*, *Lpl*, and *Cst7*). DAM primarily localized in proximity to amyloid-β plaques, showing higher expression of CD11c (encoded by *Itgax*). Loss of TREM2 blocked microglia in a homeostatic state and this resulted in increased disease severity [60], corroborating findings from patients with TREM2 variants, which confer increased risk of developing AD [61, 62]. Microglia subsets with features similar to DAM have also been described in murine models of multiple sclerosis, amyotrophic lateral sclerosis and alternative AD models, termed MGnD for microglial neurodegenerative phenotype [63]. As for DAM, the authors provided evidence that genetic loss of TREM2 resulted in a reduction of the MGnD phenotype; however, this correlated with disease amelioration, contrary to what observed by Keren-Shaul et al. [59] and in agreement with similar reports with tau models of neurodegeneration [64].

An attempt to unify the single-cell landscape of microglia in aging and neurodegeneration was carried out by Florent Ginhoux and coworkers, who, in addition to conducting their own scRNAseq analysis, also integrated protein expression data and compared their dataset to previous ones obtained from aged microglia and neurodegenerative mouse models [3]. With this integration, the authors confirmed the existence of DAM and, in addition, proposed that a cluster of microglia with an increased inflammatory and reactive phenotype is in fact of monocytic origin and accumulates with age, possibly due to increased migration into the brain, due to lapses in the blood brain barrier [3]. The authors showed that these microglia cells have a distinct phenotype that more closely resembles macrophages, indicated by the greater expression of *Il1a*, *Il1b*, *Tnf*, *Nfkbia*, *Cd49f*, *Cd54*, *Cd83*, and *Tlr* signaling genes and genes responsible for NO and ROS production. Indeed, they defined this cluster as DIM, or disease inflammatory macrophages. However, the authors did not describe clusters with increased expression of interferon-response genes, so it remains to be further clarified if DIM share any overlapping features with the OA3 and IRM clusters described earlier [50, 58].

**Fig. 2:**
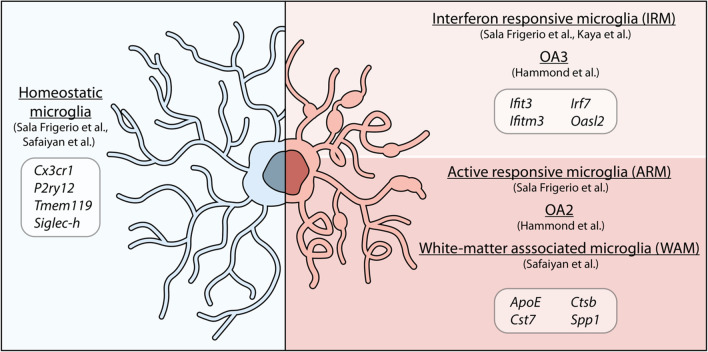
Gene signatures of aged microglia subsets. Different single-cell RNAseq studies have analyzed aged microglia. Their findings show the majority of cells to have a homeostatic gene signature, characterized by housekeeping genes that support healthy microglia function (in blue). The morphology of homeostatic microglia is highly ramified with extended and arborized processes [8]. In addition, aged mice show an enrichment of two subpopulations of microglia (in light pink, red). Although the signatures described in different studies are not completely overlapping, they share common features such as the upregulation of IFN-regulated genes (IRM, OA3) or genes related to inflammation, phagocytosis and lipid metabolism (ARM, OA2, WAM) [50, 58, 65, 67]. Aged microglia display shorter and thickened processes, with inclusions that arise from either phagocytosis or lipid accumulations [17, 134]; however, it remains to be clarified if the two subtypes have similar or distinctive morphological characteristics

Taken together, these studies suggest that aged microglia acquire a responsive “DAM-like” phenotype to deal with environmental perturbations, further confirmed by the notion that a similar phenotype is observed also in microglia from young animals during development [3]. The DAM-like cells might exert protective functions, such as increased phagocytosis of protein aggregates and lipid metabolism and deleterious ones, such as secretion of inflammatory cytokines. With age, as the number of environmental alterations increase, so does the number of “responsive” microglia. Indeed, alterations of the brain environment can also be constituted by myelin degeneration, as highlighted by Safaiyan et al., who analyzed microglia from gray and white matter by scRNAseq, showing that the greatest changes in aging concern mainly white matter microglia [65]. The highly enriched myelinated nerve fibers of the white matter are known to undergo degeneration with aging [66], leading to the accumulation of myelin debris that are typically digested by microglia [10, 65]. In addition to a commonly shared homeostatic signature, two main microglia populations were found to accumulate in the white matter with aging. The first was white-matter-associated microglia (WAM), which were linked to myelin degeneration and whose differentiation was TREM2-dependent, as reported for DAM or MGnD in AD [59, 63]. Within the white matter, microglia clustered in nodules consisting of 3–5 cells, engaged in clearing myelin debris and characterized by larger cell bodies and thick processes. At transcriptional level, WAM were defined by upregulation of lipid metabolism and phagosome-related genes (*Apoe*, *Cst7*, *Bm2*, *Lyz2*, *Cd63* and *Clec7a*), cathepsins (*Cstb*, *Ctss*, and *Ctsz*) and MHC II-related genes, further confirming their similarity to the DAM phenotype [59], of which they appear to be a precursory stage [65]. Furthermore, the authors found a second cluster of cells in white matter microglia, also shown to be TREM2-dependent and defined by the authors as “activated microglia”. This subgroup was characterized by a marked upregulation of genes related to protein synthesis, encoding ribosomal proteins and translational factors. Interestingly, this cluster increased with age also in the gray matter [65]. Although the different scRNAseq studies indicate clear overlaps, certain points remain to be further investigated, such as whether the activated microglia found by Safaiyan et al. also present an increase of IFN-response genes and whether they also identify microglia with a monocytic origin. This issue was in part addressed by the same group in a subsequent publication, in which they showed that, in aging, a greater infiltration of CD8^+^ T cells caused the induction of interferon-responsive microglia and oligodendrocytes in the white matter [67]. In this study, the authors confirmed the deleterious effect of IFN on brain health, further indicating that microglia characterized by the expression of IFN genes might contribute to neurotoxicity [67–69]. A summary of microglia states described in aging is provided in Fig. 2 and a more detailed list of overlapping genes is provided in Table 1.

**Table 1:**
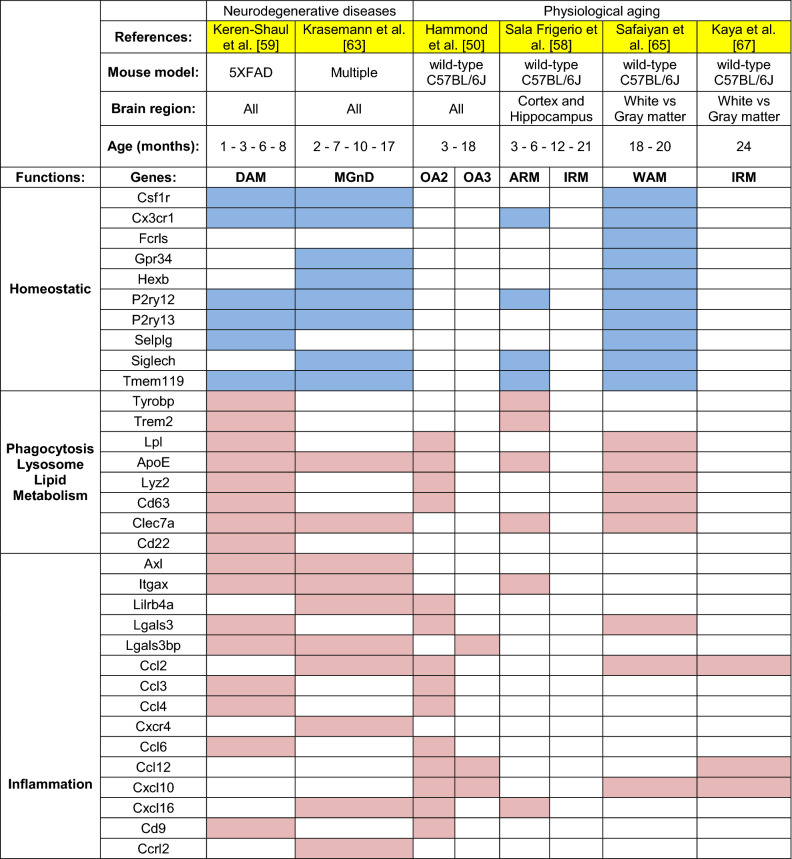

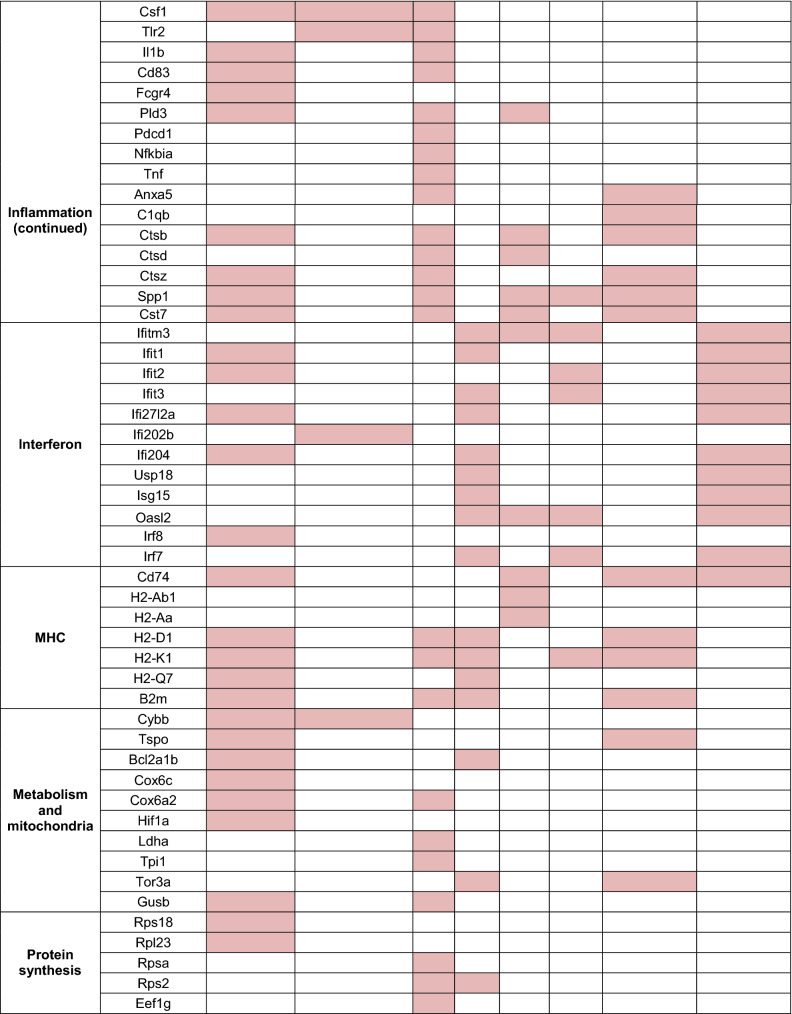
Summary of microglia genes enriched in mouse models of healthy aging and neurodegenerative diseases. Mouse microglia signatures identified in different contexts and sorted based on disease state (healthy aging/neurodegeneration), mouse models and brain areas investigated. Data shown in the table were mined from the available sources published by each respective study. For Keren-Shaul et al. [59] and Hammond et al. [50], a fold-change threshold of 1.5 (or − 1.5) was applied when mining data. Genes left as blank were below the 1.5-fold change or were not reported. For Krasemann et al. [63], Sala Frigerio et al. [58], Safaiyan et al. [65], Kaya et al. [67], genes that were not reported in the published sources were left as blank. Each gene cluster is classified according to the relative gene function and highlighted in pink, if upregulated, or blue, if downregulated, based on the enrichment analysis in each study. Genes that do not fall in the mentioned categories were excluded for simplicity. DAM, disease-associated microglia [59], MGnD, microglial neurodegenerative phenotype [63]; OA2 and OA3: Old activated microglia 2 and 3 [50]; ARM, activated responsive microglia [58]; IRM, interferon-responsive microglia [58, 67]; WAM, white-matter-associated microglia [65]

An important aspect to point out when considering these microglial states is that the expression profiles mostly rely on transcriptome studies, which might not always reflect protein expression, as reported by us [27]. Although we have a better understanding of microglia heterogeneity, the biological functions of these subsets and their contribution to brain aging remain to be properly addressed. In this respect, multi-omics approaches based on integrating multidimensional data can provide further insights in microglia diversity in aging and disease: new methods such as spatial transcriptome [70], translatome and protein analysis [71, 72], including ribosome profiling and mass spectrometry at single-cell resolution [73–75], could significantly help expand our knowledge.

### Are aged microglia senescent?

Many of the altered characteristics described in aged microglia could be attributed to senescence; indeed, different groups have studied whether aged microglia phenotypes truly correspond to the definition of senescent cells.

Cellular senescence is characterized by an irreversible cell-cycle arrest mediated by cyclin-dependent kinase inhibitors (Cdkn2a/p16^INK4^, Cdkn1a/p21^WAF1/Cip1^), accompanied by macromolecular and organelle damage (for a systematic review of senescence and its features see Gorgoulis et al. [76]). Senescent cells are characterized by telomere shortening, DNA defects, proteotoxicity and a deregulated metabolic profile due to mitochondrial dysfunction and lysosomal changes. Importantly, senescent cells are also characterized by a senescence-associated secretory phenotype (SASP), which affects surrounding cells by secretion of inflammatory mediators such as TNF, IL-6, chemokines and remodeling factors [76]. Senescence has been linked to organismal aging, as a factor contributing to age-related dysfunctions, nonetheless, it can also be induced in younger tissues by stressful insults, leading to distinctive secretory phenotypes [77]. In microglia, different phenotypes based on sex and brain regions have been reported. An age-dependent increase of senescence-related genes and higher expression of Cdkn2a/p16^INK4^ was found in particular in the hippocampus of old female mice [78]. The highest frequency of p16-positive brain cells was found in microglia (*Tmem119*^+^) and monocyte (*Ly6c2*^+^) populations. Transcriptome profiling of these two p16-positive myeloid clusters showed an upregulation of senescence-related genes (*Cdkn2a*, *Cdkn1a*, and *Blc2*), chemoattractant SASP factors (*Ccl2*, *Ccl5*, and *Spp1*), MHC genes and lysosomal stress markers (*Lgals3* and *Lgals3bp*) [78]. This population also showed an overlapping gene expression profile characteristic of DAM (*Apoe*, *B2m*, *Cst7*, *Fth1*, *Iftm3*, *Itgax*, and *Lpl*), in parallel with a downregulation of homeostatic genes (*Cx3cr1*, *Hexb*, *Marcks*, *P2ry12*, *Selplg*, and *Tmem110*) [78]. Interestingly, the expression pattern of this cluster assessed by mass cytometry resembled a microglia subpopulation with high levels of CD11c and MHC II molecules (CD45^+^CD11b^+^CX3CR1^lo^MHCII^hi^CD206^lo^CD11c^+^CD103^lo^), which expand in an age-dependent manner [78].

The contribution of p16^INK4^-positive cells to the aging phenotype was then assessed by whole-body ablation of highly-expressing p16 cells using the *INK-ATTAC* mouse model, in which administration of the drug AP20187 induces selective apoptosis through the dimerization of a membrane-bound FK506-binding-protein–caspase 8 (FKBP–Casp8) fusion protein, specifically expressed under the p16^INK4^-gene promoter, as first described by Baker et al. [79]. Systemic ablation reduced p16^+^-microglia only in female mice and not male counterparts, nonetheless, the microglia subsets that progressively expand in aging remained largely unaffected by the treatment [78]. More generally, p16^+^ cell-targeting led to a much broader effect, that is a reduction of infiltrating immune cells, especially B and T cells [78, 80], which have been reported to accumulate in the aging brain in mice [81–83] and humans [84]. This might depend on the direct effect of removing infiltrating senescent immune cells in *INK-ATTAC* mice, as well as the resulting reduced expression of SASP-associated chemoattractants. A similar trend was also observed in a previous study, but carried out in male mice [80]. Here, the highest number of p16^INK4^-positive cells accumulating in aging were represented by both oligodendrocytes progenitor cells (OPCs) and aged microglia, which positively correlated with higher expression of inflammatory genes involved in immune cell activation (*Il1b*, *Mif*, *Csf1*, *Axl*, *Csf2ra*, and *Timp2).* The frequency of p16^INK4^-positive microglia and infiltrating CD3^+^ immune cells also decreased in *INK-ATTAC*, AP20187-treated male mice in aging, but there was only a mild reduction in the production of inflammatory molecules in whole brain homogenates. Even though sex accounted for different age-related signatures in the aging hippocampus [85], these studies showed that systemic clearance of p16-positive cells improved cognitive functions in aging [78, 80], as well as in neurodegenerative conditions, as recently shown in a mouse model of tau-mediated disease [86]. In line with previous findings, spatial-resolved transcriptome analysis in brain sections of aging animals confirmed the age-dependent increase of Cdkn2a expression [87]. Moreover, the microdomains containing senescent cells heterogeneously distributed in the white matter, cortical gray matter and hippocampus and were mainly represented by cluster of cells expressing microglia, endothelial, oligodendrocyte and OPCs markers [87].

An alternative approach, in which p16^high^-expressing cells were isolated based on the expression of a reporter gene under the control of the p16 promoter, showed that aged microglia represented the majority among brain cells [88]. In subclustering analysis, p16^+^-microglia mostly concentrated in two unknown and distinct clusters (UM1 and UM2), although they were also found in clusters associated with DAM and interferon-response signatures. Although UM1 and UM2 had greater p16 expression, the senescence-associated genes were more enriched in the DAM cluster, indicating a non-linear correlation between p16 and senescence [88].

Further investigations are required to clarify the extent and role of senescence in microglia aging, especially how it affects microglia heterogeneity in different brain areas. Furthermore, since clearance of p16^high^-expressing cells in *INK-ATTAC*, AP20187-treated mice is not restricted to a specific cell type (i.e., microglia) but affects the whole body, cell-specific targeting strategies, in combination with or without senescence-targeting transgenic mouse models [79, 89, 90], would help clarify the specific role of senescent microglia. Finally, it is important to point out that, although high expression of p16 is broadly recognized as a marker of senescence, not all senescent cells express p16 and its expression alone is not sufficient to indicate a senescent state. For example, alginate-encapsulated senescent cells injected intraperitoneally in young mice were mostly surrounded by a F4/80^+^ macrophage subpopulation with an increased expression of p16^INK4^ and senescence-associated beta-galactosidase (SaβG). The upregulation of these two well-known senescence markers was due to a physiological response to stimulation and it occurred in a p53-independent manner, which is otherwise required to modulate cellular senescence and organismal aging [91–93]. Based on these points, a senescence state in aging cannot uniquely be defined by p16 alone but should take into account additional senescent markers.

### Microglia numbers and contribution from circulating monocytes

Earlier reports indicated that microglia numbers increased with age [18]; however, subsequent studies have reported divergent results [94, 95]. A more recent study took a systematic approach to assess microglia cell numbers, proliferation and turnover, both in mice and humans [96]. The authors used pulse-chase experiments to show that the number of microglia remained constant also in old age. Nonetheless, microglia underwent multiple rounds of proliferation, more than previously evaluated [4], and their number was maintained constant by a balance of proliferation and apoptosis [96]. The authors concluded that circulating monocytes did not contribute to the microglia pool over time, since the number of microglia cells was similar in aged WT and CCR2 KO mice, which possess significantly reduced circulating monocytes [96]. However, this is in contrast to what was reported by Silvin et al. [3]. As mentioned earlier, in this study, the authors found that a proportion of microglia, which they define as disease inflammatory macrophages (DIM), have a monocyte origin and increase with age, expanding even further in mouse models of neurodegeneration. With bone marrow chimeric mice, they could show that monocytes derived from transplanted Ms4a3CreRosaTomato-expressing cells were found in the brain and recapitulated the DIM phenotype [3]. Nonetheless, previous publications have questioned results obtained with bone marrow chimeras, pointing out that irradiation can cause depletion of microglia in the brain, thus inducing repopulation also from peripheral monocytes [97].

### Microglia and age-related alterations in the brain lymphatics system

The discovery of meningeal lymphatics revealed new concepts of lymphatic drainage in the brain, which plays a central role in draining protein aggregates and, therefore, is particularly important in aging and neurodegeneration [98, 99]. In addition, the meningeal lymphatics facilitate immune-cell trafficking in the brain, prompting a review of the old concept that the CNS is an immune privileged site, which originated from past experiments showing that tissue transplanted into the brain was not rejected [100]. Like in other tissues [101], lymphatic drainage in the brain declines with age and is accompanied by decreased meningeal lymphatic vessel diameter and diminished drainage of cerebrospinal fluid into the deep cervical lymph nodes [102]. Furthermore, gene expression analysis of lymphatic endothelial cells (LECs) from the meninges further indicated that they are functionally impaired in aging [102]. Destruction of meningeal lymphatics in young mice resulted in impaired brain perfusion by the cerebrospinal fluid and resulted in learning and memory deficits. Conversely, cognitive functions of old mice could be improved by increased expression of VEGF-C, which caused improved lymphatic diameter and drainage. Therefore, age-dependent decline in lymphatic drainage of the brain contributes to cognitive decline. In a subsequent study, the Kipnis group further assessed brain drainage and lymphatics in AD, revealing how microglia are directly affected by impaired brain drainage [103]. By analyzing the transcriptional profile of mouse meningeal LECs, microglia and brain blood endothelial cells, the authors showed that meningeal lymphatic function is coupled to microglia activation in AD: ablation of meningeal lymphatics in 5xFAD animals exacerbated plaque deposition, microgliosis and neurovascular alterations. Analysis of homeostatic and DAM gene signatures in microglia from 5xFAD mice with ablated meningeal lymphatics revealed higher expression of *Apoe*, *Lyz2*, *Fth1*, *Timp2*, *H2-d1*, *Axl*, *Cst7*, *Spp1* and *Lpl*, genes that are characteristic of the DAM phenotype, and further reduced expression of homeostatic genes, suggesting that “DAM-like” microglia observed in aging could be caused by changes in the brain lymphatics [103]. Interestingly, changes caused by worsening lymphatic drainage could be reversed by exercise, further underscoring the importance of efficient lymphatic drainage in the context of amyloid-β deposition [104]. It remains to be further elucidated if alterations in lymphatics health and CNS drainage, independently of any amyloid-β deposition, cause potentially detrimental alterations in microglia. It is known that CNS drainage decreases with age in human subjects [105] and that a correct function of the brain lymphatic system depends on sleep, which is known to be altered in aging [106]. Nonetheless, it would be interesting to assess to what extent brain lymphatic drainage varies in older individuals, if it is affected by lifestyle factors or comorbidities and to what extent microglia mediate the deleterious effect of reduced lymphatics drainage. Exploring these questions will be important in determining whether modulation of meningeal lymphatic function in aged individuals could represent a novel therapeutic strategy in age-related cognitive decline and if these therapeutic strategies would need to take microglia into account.

### Interplay of microglia, astrocytes and oligodendrocytes in aging

In the brain, microglia constitute one component of a complex network of glial cells, which includes astrocytes and oligodendrocytes, the brain macroglia [107].

Astrocytes are the most abundant neuroglial subtype in the brain. The main functions of astrocytes are maintaining ion homeostasis, supporting neurotransmitter transmission, secreting growth factor, remodeling synapses, regulating oxidative stress and maintaining the integrity of the blood brain barrier, since their endfeet directly interact with brain vessels [108]. Astrocytes and microglia regulate each other's state and function by releasing specific cytokines and growth factors. Depending on the physiological setting, they can work either synergistically or antagonistically; activated microglia induce the generation of neurotoxic astrocytes by secretion of IL-1a, TNF and C1q [109]. On the other hand, microglia inhibit astrocytic engulfment of synapses, in a TREM2-dependent fashion [110]. Microglia and astrocytes also secrete factors to maintain their homeostatic state; microglia-derived IL-10 binds to IL-10 receptor on astrocytes, causing them to secrete TGF-β [87], a critical factor in the maintenance of microglia homeostatic phenotype [111].

In aging, alterations in microglia and astrocytes exert a reciprocal influence on their phenotype. Transcriptome analysis of aged astrocytes indicated that they acquired a more reactive phenotype [112] and altered their metabolism [113], including down regulating cholesterol biosynthesis, which is required to maintain the microglia homeostatic phenotype [114]. Furthermore, aged astrocytes did not increase their expression of IL-10R or secretion of TGF-β upon a peripheral LPS challenge, failing to exert their inhibitory feedback on microglia. Indeed, hippocampal microglia of aged mice had an amplified and prolonged neuroinflammatory response compared to young mice, further exacerbated in mice with IL-10 receptor KO astrocytes [113]. A different study, using the Bac-Trap method to perform RNA sequencing of astrocytes from different brain regions at different ages, also revealed that aged astrocytes were prone to a reactive neuroinflammatory phenotype. Interestingly, hippocampal and striatal astrocytes upregulated reactive genes more strongly compared with cortical counterparts. The aging-induced upregulation of reactive astrocyte genes was significantly reduced in IL1α; Tnf; C1q knockout mice. These three factors are released by activated microglia in the CNS, hence this indicated that activated microglia in turn promote astrocyte hyperresponsiveness in normal brain aging [115]. Pan et al. performed RNAseq analyses on microglia and astrocytes freshly isolated from WT and APP-PS1 mice brains at five different ages. They found that inflammatory cytokines secreted by microglia appeared earlier than the upregulation of neuroinflammatory genes in reactive astrocytes, further indicating that microglia might induce activation of astrocytes in aging and AD progression [116]. The aging-induced upregulation of reactive genes by astrocytes could contribute to the greater vulnerability of the aged brain to injury in neurodegeneration [116], LPS challenge [115] and blood brain barrier disfunction [117].

The other macroglia cells of the brain are oligodendrocytes, the myelinating glia. They are closely connected with neurons and produce myelin sheaths, which envelop axons and protect their structural integrity, accelerating nerve impulse conduction and offering trophic support [118]. Oligodendrocytes are mainly differentiated from oligodendrocyte precursor cells, OPCs. When myelin is damaged, OPCs are activated and proliferate, migrate and finally differentiate into mature oligodendrocytes in large numbers [119]. Oligodendrocytes/OPCs and microglia influence each other; on the one hand, activated microglia produce various pro-inflammatory mediators including matrix metalloproteinases, lipolytic enzymes, reactive oxygen and nitrogen species, excitotoxins, chemokines and cytokines, which can be harmful to oligodendrocytes. On the other hand, microglia are necessary for the proliferation and maturation of OPCs into a demyelinated area for wound healing, regeneration and repair in the CNS [120]. In turn, oligodendrocytes can control microglial activity by producing chemokines, cytokines, and chaperonins [120]. Communication between the oligodendrocytes/OPCs and microglia is maintained in the aging brain but can be altered due to the altered microglia phenotype or to myelin dysfunction. The extracellular matrix (ECM) is a critical factor regulating OPC differentiation during remyelination [121]. Baror et al. showed that microglia increased the expression of the proteoglycan NG2 with age and, in doing so, could divert differentiation of OPCs into astrocytes in vitro [122]. Interestingly, changes in aging microglia were caused by exposure to high levels of TGF-β: with aging, the levels of circulating TGF-β increased, causing a decline in remyelination by impairing the ability of microglia to promote oligodendrocyte differentiation from OPCs [122]. Luan et al. showed that aged mouse brains presented demyelination and that OPCs in the basal state were significantly increased [123]. At the same time, however, the OPCs that underwent differentiation to mature oligodendrocytes were greatly decreased. Cell–cell interaction analysis showed that activated microglia in the aging brain released inflammatory factors that suppressed OPC differentiation. In this context, inhibition of Stat1 in aged microglia could induce a switch to a tissue repair phenotype that promoted oligodendrocyte generation [123].

More recent evidence indicates that microglia are dispensable for OPC maturation and differentiation, nonetheless they are critical to maintain myelin health by preventing demyelination and hypermyelination [10], confirming microglia’s role in preserving myelin integrity that is altered in aging.

### Aged microglia and the gut–microbiome–brain axis

Despite the anatomical separation between the CNS and the gastrointestinal tract, a bidirectional connection between the two systems has been reported and defined as the ‘brain-gut axis’ [124]. It is known that the microbiome influences immune cells [125] and more recent studies have demonstrated that also microglia are sensitive to factors produced by the gut microbiota, which can alter microglial numbers, size, transcriptome, and surveillance functions. Furthermore, it appears that changes in the microbiome associated with aging might be linked to alterations in microglia (reviewed in [126]), as demonstrated by the finding that germ-free (GF) mice displayed global defects in microglia but recolonization of microbiota or microbiota-derived products could reverse defects of GF microglia morphology, rescuing their immature phenotype. The main factors responsible for the effect on microglia were short-chain fatty acids (SCFAs). Indeed, microglia lacking the short-chain fatty acid receptor FFAR2 presented similar defects to GF microglia [127]. Nonetheless, subsequent studies have shown that SCFAs play a detrimental role on microglia in the context of neurodegenerative diseases, such as Parkinson’s disease [128] and Alzheimer’s disease [128], exacerbating disease progression. In aging, a new crosstalk between the altered aged microbiome and microglia was revealed by Mossad et al., using RNAseq to analyze FACS-purified microglia from the whole brain of young (6–10 weeks) and aged (96–104 week) mice, housed under GF or (control) specific pathogen-free (SPF) conditions [129].

Transcriptomic differences in microglia from GF and SPF mice were more prominent at older age: as expected, microglia from SPF mice presented the strongest alterations typical of aged microglia, such as upregulation of inflammatory, IFN signaling and oxidative stress genes, including *Hif1a*, as well as morphological defects. On the other hand, microglia of aged GF mice retained a hyper-ramified morphology and presented a less pronounced aged transcriptional phenotype. In microglia from (control) SPF mice, increased oxidative stress correlated with impaired mitochondrial activity and ATP production, which were not observed in GF mice, indicating that the aged microbiome is affecting microglia bioenergetics in aging. In terms of altered metabolites in aging, among the short-chain fatty acids, acetate and propionate were found increased; however, unbiased metabolomic analyses of serum and brain tissue in young adult and aged SPF mice revealed that the factor responsible for microglia “aging” was N6-carboxymethyl lysine (CML), an advanced glycation end product (AGE). This metabolite accumulated in the blood and brain of aged SPF mice and could directly induce the altered microglia phenotype when administered in young mice, resulting in an increase in ROS, impaired mitochondrial activity and a decrease in the ATP reservoir. Nonetheless, increased CML blood levels (and relative uptake by microglia) were not due to alterations in the aged microbiome, since CML in fecal pellets of aged GF mice was actually higher than in aged SPF mice. The reason behind the increased blood levels of CML was rather the increased permeability of the gut barrier in SPF conditions. Indeed, colonization of young mice with an aged microbiome resulted in overall increased intestinal permeability [129]. Taken together, these results show that alterations in the microbiome affect different physiological functions that ultimately impact microglia aging and suggest potential preventive avenues that deserve further investigation.

## Morphology and phenotype of human microglia in aging

Whereas microglia from animal models, especially rodents, have been extensively investigated, studies of human microglia were more limited in the past. However, recent advances in microglia isolation and sequencing techniques have resulted in a number of studies of bulk or single cells (sc)/single nuclei (sn) RNAseq of human microglia. Despite the increase in published reports, some caveats remain, such as the relatively small number of human samples investigated [130], especially in terms of comparison of young versus old individuals, as well as issues associated with the isolation of cells or nuclei and purification of mRNA, which results in relatively smaller RNA datasets [131]. Indeed, some publications have highlighted the inherent technical challenges and questioned the validity of, for example, single nuclei RNAseq to investigate human microglia phenotypes [132].

This may be the reason why different human RNA datasets seem to share limited overlap [130, 131, 133]. More importantly, it remains to be established if there is any overlap with mouse aged microglia, or if this is indeed rather limited, as some of these studies indicate [131, 133].

A first comprehensive study of human microglia gene expression was carried out by Galatro et al. [133], who assessed the transcriptome of 39 human non-demented cortex samples, with an age range from 34 to 102. Hierarchical clustering of the most differentially expressed genes resulted in two separate clusters based on age, indicating that a sufficient number of genes were differentially expressed with age.

Nonetheless, the number of altered genes was relatively limited (= 212 upregulated and 360 downregulated). The most differentially expressed were genes regulating actin dynamics and cell adhesion, confirming previous findings in aged microglia morphology in rodent and human microglia [17, 134].

Notably, the authors compared their dataset to changes happening in mouse microglia with age and they found a very limited number of overlapping genes, 14 upregulated and 9 downregulated, mostly converging on the biological pathway “positive regulation of cell–matrix adhesion”.

In any case, this study highlighted that a great number of genes were expressed in human microglia but not mouse microglia, including genes related to peptide trimming for MHC antigen presentation (*NLRC5* and *CIITA*), genes regulating retrovirus replication and retrotransposon mobility (*APOBEC3C*) and sialic acid-binding immunoglobulin-like lectins (Siglecs), such as *CD33*, which have an inhibitory role through their immunoreceptor tyrosine-based inhibition motif (ITIM). Overall, the human-specific microglia genes suggested a greater regulation of immune responses compared to mouse counterparts; however, none of these genes appeared to be differentially expressed with age [133].

A more recent study by Olah et al. [130] presented a limited overlap with data generated by Galatro et al. [133]. In Olah et al., pathways enriched in human microglia with aging were linked to DNA damage, telomere maintenance and phagocytosis regulation; however, the study assessed only a small number of samples, 10, of which only 3 were middle aged. A direct comparison of middle-aged vs old microglia indicated that the top upregulated pathways were linked to amyloid fiber formation and the top downregulated ones to TGF-β signaling. The first observation is not surprising, since the older individuals assessed in this study were non-demented but, nonetheless, positive (9 out of 10) for amyloid. On the other hand, loss of TGF-β signaling further corroborated the notion that microglia lose their homeostatic state with aging, as emerged from mouse studies [44, 45]. Nonetheless, loss of TGF-β signaling is perhaps not surprising, given the amyloid-β deposition that characterized the samples analyzed.

In addition, this study highlighted that aged microglia have increased DNA damage and telomere maintenance responses, corroborating previous studies by Streit and coworkers, who first described shorter telomeres in aging human microglia, proposing replicative senescence as its cause [135, 136]. Furthermore, the Streit group carried out morphological analysis of human aged microglia, describing in details their dystrophic morphology [134], characterized by alterations in their cytoplasmic processes such as deramification and thinning or fragmentation, presence of spheroids and gnarling (for a schematic representation, see Fig. 2). It remains to be further clarified if microglia presenting these characteristics are the same identified by Galatro et al. as cells with reduced actin dynamics [133], which might be responsible for the dystrophic morphology observed by histology. Streit and coworkers also described dystrophic microglia to accumulate ferritin, the intracellular protein that stores iron, hypothesizing that altered iron metabolism was contributing to their phenotype [137, 138]. Alterations in iron metabolism have not been highlighted in human RNAseq studies, nonetheless, it is possible that either these alterations were not among the most highly differentially expressed genes or that changes are caused mainly by posttranscriptional mechanisms. Interestingly, iron and ferritin accumulation were found also in mouse models of aging and neurodegeneration [139, 140], further highlighting how impaired iron metabolism might be an important factor contributing to the altered aging microglia phenotype, both in rodents and humans.

A more recent publication by de Paiva Lopes et al. [141] has assessed expression Quantitative Trait Loci (eQTLs) from 255 human microglia samples isolated from 4 different brain regions of 115 individuals, of which one-fourth was healthy or non-demented controls, while the rest were from neurodegenerative, neurological and neuropsychiatric patients. Interestingly, this study did not identify differentially expressed genes between male and female individuals, whereas the strongest variance was associated with age. Concerning the different brain areas, they accounted for little variance overall, although a small subset of genes was strongly variable between regions, with greatest differences found between the subventricular zone and cortical regions. Gene clustering identified four clusters, with cluster 1 containing genes downregulated in AD brains and in response to in vitro culture; indeed, these genes corresponded to homeostatic functions. Cluster 2 contained genes implicated in inflammatory processes and genes upregulated in AD. Cluster 3 contained genes downregulated in the subventricular zone compared to other regions, such as *CX3CL1*, *CCR2* and *FCGR3B*. Finally, cluster 4 contained genes related to hormone signaling and interferon response [141]. This constituted the first evidence for enrichment of IFN signaling in human microglia, which has been described in mouse microglia in aging [31, 50, 58, 142]. The reason why it was not detected in other studies of human microglia might vary, it could be due to limitations of the isolation and sequencing techniques, microglia regional heterogeneity or technical limitations of single-cell studies. Consistent with Galatro et al. [133], de Paiva Lopes et al. also reported downregulation of cell motility, IL-6 signaling and antioxidant defenses with age, while the upregulated pathways included phagosome formation, lipid metabolism and IFN signaling. Increased phagocytosis and lipid metabolism have been described in activated responsive microglia (ARM) [58] and white-matter-associated microglia (WAM) [65], indicating that, as described in mice, also human aged brains are characterized by an expansion of the microglia subset that present an activated phenotype and is responding to environmental perturbations, such as myelin dysfunction or amyloid-β deposition, since some of the healthy controls in human studies were non-demented subjects presenting amyloid deposition. Alongside these responsive subsets, a microglia subset defined by upregulation of IFN signaling is observed also in aged human brains, further corroborating the notion that these cells might exert a more neurotoxic role [68, 69]. Nonetheless, despite these overlaps between mouse and human microglia aging phenotypes, many differences remain, underlying the importance to fully characterize mouse/human microglia phenotypes as well as use additional models that can more closely recapitulate the characteristics of human microglia in human brains. One such method could be the use of human iPSC-derived microglia (iPSC-MG). Since the first report on the generation of microglia-like cells from human pluripotent stem cells [143, 144], many studies have successfully generated and analyzed iPSC-MG. Functional assays and RNAseq experiments have shown that iPSC-MG recapitulate the main features of microglia isolated from human brain [143–146]. However, recapitulating the human microglia aging phenotype remains a challenge. One way to address this issue was to use xenograft experiments in which human iPSC-MG were transplanted into mouse brains, as reported by Hasselmann et al. [147]. One day after birth, the authors transplanted human iPSC-derived hematopoietic progenitor cells (HPC) into the brains of humanized 5xFAD mice, a mouse model of amyloid-β deposition. Analysis at 9 months of age showed that the injected iPSC-HPC differentiated into functional microglia inside the murine brain and adopted a DAM-like phenotype in response to amyloid plaques. Importantly, however, the study indicated only a partial overlap between mouse and human microglia in the expression of genes that confer susceptibility to neurodegenerative diseases, further highlighting the importance of using alternative and relevant models.

Progress has been made also in recapitulating different microglia phenotypes in vitro, as recently described by the Kampmann lab [148], who showed that microglial heterogeneity can be induced by targeting 39 genes in iPSC-MG using CRISPRi. Some of the resulting clusters partially resemble microglial diversity in aging, such as clusters that strongly express interferon-induced genes or a SPP1+ cluster that exhibits a DAM-like phenotype.

In conclusion, iPSC-MG offer an alternative promising tool to study human microglia and manipulate their responses.

## Concluding remarks

Both rodent and human aging microglia are characterized by alterations in morphology, phagocytosis, metabolism and inflammatory phenotype, which appear to play protective and detrimental roles in maintaining brain homeostasis and preserving their ability to respond to non-sterile and sterile insults. Furthermore, more recent evidence indicates that environmental factors, such as meningeal lymphatics health and production of metabolites from the gut microbiome, can affect brain homeostasis by affecting microglia reactivity and phenotype.

The recent scRNAseq studies suggest that different subsets of microglia already exist in young adults; however, they expand in aging and even more so in neurodegeneration. Nonetheless, we still do not know the full extent of microglia plasticity and how firm these phenotypes are [149]. Furthermore, we still need a comprehensive analysis of genes and functions that are specific to human microglia and cannot be modeled in rodents. Future studies will need to better elucidate microglia plasticity and the peculiarities of human microglia, in order to pave the way for the development of new strategies to prevent or treat age-related diseases. In this respect, human iPSC-derived microglia can offer an interesting tool that remains to be further explored.

## Data Availability

Not applicable.
